# CCL25/CCR9 Interactions Regulate Large Intestinal Inflammation in a Murine Model of Acute Colitis

**DOI:** 10.1371/journal.pone.0016442

**Published:** 2011-01-25

**Authors:** Marc-Andre Wurbel, Maria G. McIntire, Peter Dwyer, Edda Fiebiger

**Affiliations:** 1 Division of Gastroenterology and Nutrition, Children's Hospital Boston, and Department of Pediatrics, Harvard Medical School, Boston, Massachusetts, United States of America; 2 Department of Pathology, Harvard Medical School, Brigham and Women's Hospital, Boston, Massachusetts, United States of America; Emory Unviersity, United States

## Abstract

**Background & Aims:**

CCL25/CCR9 is a non-promiscuous chemokine/receptor pair and a key regulator of leukocyte migration to the small intestine. We investigated here whether CCL25/CCR9 interactions also play a role in the regulation of inflammatory responses in the large intestine.

**Methods:**

Acute inflammation and recovery in wild-type (WT) and CCR9^−/−^ mice was studied in a model of dextran sulfate sodium (DSS)-induced colitis. Distribution studies and phenotypic characterization of dendritic cell subsets and macrophage were performed by flow cytometry. Inflammatory bowel disease (IBD) scores were assessed and expression of inflammatory cytokines was studied at the mRNA and the protein level.

**Results:**

CCL25 and CCR9 are both expressed in the large intestine and are upregulated during DSS colitis. CCR9^−/−^ mice are more susceptible to DSS colitis than WT littermate controls as shown by higher mortality, increased IBD score and delayed recovery. During recovery, the CCR9^−/−^ colonic mucosa is characterized by the accumulation of activated macrophages and elevated levels of Th1/Th17 inflammatory cytokines. Activated plasmacytoid dendritic cells (DCs) accumulate in mesenteric lymph nodes (MLNs) of CCR9^−/−^ animals, altering the local ratio of DC subsets. Upon re-stimulation, T cells isolated from these MLNs secrete significantly higher levels of TNFα, IFNγ, IL2, IL-6 and IL-17A while down modulating IL-10 production.

**Conclusions:**

Our results demonstrate that CCL25/CCR9 interactions regulate inflammatory immune responses in the large intestinal mucosa by balancing different subsets of dendritic cells. These findings have important implications for the use of CCR9-inhibitors in therapy of human IBD as they indicate a potential risk for patients with large intestinal inflammation.

## Introduction

Crohn's disease and ulcerative colitis represent two clinical subtypes of inflammatory bowel disease (IBD). Infiltration of inflammatory leukocytes into the gastrointestinal mucosa critically regulates development as well as progression of both types of IBD. Tissue- and cell type-specific leukocyte trafficking is orchestrated by chemokines and their receptors. Interaction of CCL25 with CCR9 identifies one of the few non-promiscuous chemokine/receptor pairs involved in gut-specific migration of leukocytes. CCL25 is strongly expressed by the small intestinal epithelium [1], [2], [3] and regulates trafficking of gut-specific memory/effector T cells via upregulation of the integrin homing receptor α4β7 and CCR9 [4], [5], [6], [7]. The chemokine itself is dispensable for the development of CCR9^high^ intestinal memory-phenotype T cells. CCX282-B (Traficet-EN, ChemoCentryx) is a small molecule that can antagonize CCR9 function and has already shown promising results in a phase II clinical trial of moderate to severe Crohn's Disease patients as it successfully delays disease progression [8]. As CCL25/CCR9 interactions are currently considered dispensable for the regulation of inflammation in the large intestine, the therapeutic effect of the CCR9-antagonist has not been studied in ulcerative colitis.

Several different murine models have been described to study the pathophysiology of ulcerative colitis [9]. Administration of Dextran Sulfate Sodium (DSS) in drinking water induces a reversible form of colitis in mice [10]. This model is characterized by acute tissue inflammation in the colon and mimics the pathology of human ulcerative colitis. Since immuno-compromised mice such as Recombinase-Activating Gene (RAG) deficient mice or severe combined immune deficiency (SCID) mice lacking the T and B cell compartment remain susceptible in this disease model [11], a central role for monocytes/macrophages (MΦ) and dendritic cells (DCs) was proposed for the pathogenesis of DSS colitis and likewise for human ulcerative colitis. Consequently, inflammatory MΦ were described as antigen-presenting cells (APCs) that activate T cells and induce T cell proliferation in DSS colitis [12], [13].

MΦ and intestinal DCs are located in the intestinal lamina propria (LP), Peyer's patches (PPs) and mesenteric lymph nodes (MLN) and are the first APCs to sense and respond to exogenous antigens or tissue injury [14]. Several subpopulations of DCs and MΦ have been described in the intestinal microenvironment [15], [16], [17]. Their respective functions for the induction of protective immunity versus immune tolerance in the gut remain poorly understood [14]. Recent studies have shown that depletion of DCs during DSS-induced colitis leads to ameliorated or exacerbated symptoms depending on the time point of intervention [18], [19], [20]. These observations imply that concerted trafficking of different DC subsets is critical for onset and recovery of colitis. CCR9 is a candidate chemokine receptor for the regulation of this type of DC trafficking, because it has been demonstrated that CD11c-positive splenic DCs as well as peritoneal MΦ migrate towards a CCL25 chemotactic gradient [1]. However the role of CCL25/CCR9 interactions during the regulation of large intestinal inflammation has not been studied so far.

Here we describe a so far unanticipated role of CCL25 and CCR9 in the regulation of large intestinal inflammation. We show that CCR9^−/−^ animals display exacerbated colitis. In the absence of physiological CCL25/CCR9 interactions, we detect an imbalance of DC subpopulations and the accumulation of inflammatory MΦ in the lamina propria of the colon and in gut-associated lymphoid tissue.

## Materials and Methods

### Mice

The generation of CCR9^−/−^ and CCL25^−/−^ mice has been described previously [7], [21]. All strains of mice, including C57Bl/6 control mice were bred in the animal facility at Children's Hospital Boston, born and held in the same room under specific pathogen-free conditions. To avoid variations of commensal bacteria in our experiments, littermate controls were used. All animal experiments were approved under animal protocol number 09-03-1326 *by Children's Hospital Boston Institutional Animal Care and Use Committee. Children's Hospital Boston's assurance number is A3303-01. Children's* Hospital Boston is accredited by AAALAC International. All efforts were made to minimize suffering of animals.

### Dextran Sulfate Sodium- (DSS) mediated colitis

Sex- and age-matched mice received 2% DSS (36–50 kDa, MP Biomedicals, LCC, Solon, OH) in the drinking water for 7 days followed by 10-day water administration. The animal weight was recorded daily. For flow cytometry, histology and mRNA analysis, mice were euthanized at indicated time points, with day 0 (d0) corresponding to the initiation of DSS treatment. All efforts were made to minimize suffering of animals.

### Preparation of cell suspensions

Spleens (SPL) and mesenteric lymph nodes (MLN) were harvested in HBSS with Ca^2+^ and Mg^2+^ supplemented with 2% fetal calf serum (FCS) and 10mM HEPES. SPL and MLN suspensions were obtained by collagenase VIII digestion (Sigma-Aldrich, St. Louis, MO) for 30 min at 37°C. Lamina Propria Lymphocyte (LPL) suspensions were obtained as previously described [7]. Large intestines were flushed and opened longitudinally. To remove intra-epithelial lymphocytes and epithelial cells, intestinal pieces were incubated in HBSS without Ca^2+^/Mg^2+^ supplemented with 10mM EDTA, 10mM HEPES, 0.5% FCS and 1.5 mM DTET, for 2×20 min at 37°C. Intestinal pieces were digested in HBSS with Ca^2+^/Mg^2+^, 20% FCS, 100 U/ml collagenase VIII and 5 µg/ml DNase (Sigma-Aldrich) for 60–90 min at 37°C. LPL were purified over a 40%–100% Percoll gradient (GE Healthcare Bio-Sciences Corp, Piscataway, NJ).

### Flow Cytometry Analysis

Cell suspensions from SPL, MLN and large intestinal LP were analyzed by flow cytometry. Cells were stained with FITC-, PE-, PerCP-Cy5.5, PE-Cy7-, APC- or AF-647, APC-Cy7- or APC-eF780-labeled mAbs for MHC II, CD11c, CD11b, Ly6C, PDCA-1, CD103, CD8α, B220, CD19 (BD Biosciences, Franklin Lakes, NJ), CCR9 (eBioscience, San Diego, CA), DX5 and CD3ε (Biolegend, San Diego, CA). Blocking of FcγR-binding was performed using mouse and rat serum (Jackson Immunoresearch, West Grove, PA). Cells were analyzed on a FACS Canto II (BD Biosciences, Franklin Lakes, NJ). Data were collected using FACS Diva software (BD Biosciences) and analyzed with FlowJo software (TreeStar, Ashland, OR).

### Histology and IBD scoring

Intestinal samples of the distal colon were harvested, fixed in 10% formalin and embedded in paraffin for H&E staining. Histological IBD scoring was performed blindly by a pathologist as follows: IBD scores corresponding to 0 = normal, 1 = mild, 2 = moderate, 3 = severe were attributed to activity grade, changes of crypt architecture, basal lymphoplasmacytosis, expansion of lamina propria and epithelial hyperplasia. Scores were graphed in a total range of 0–15.

### mRNA quantification

Total RNA was extracted with TRIzol™ (Invitrogen, Carlsbad, CA) according to the manufacturer's guidelines. Quantification of total RNA was performed with a Nanodrop™ spectrophotometer (Thermo Scientific). For real-time RT-PCR, cDNA was synthesized with iScript™ Select cDNA Synthesis kit (Biorad, Hercules, CA). Amplification was performed with the iQ5 quantitative PCR System (Biorad) and iQ™ SYBR® Green Supermix (Biorad) on 50ng RNA. CCR9 and CCL25 mRNAs were quantified using β-actin mRNA for normalization. Primer sequences: CCL25 Forward: 5′-ccaaggtgcctttgaagact-3′, CCL25 Reverse: 5′-tcctccagctggtggttact-3′, CCR9 Forward: 5′-ccaggaaatctctggtctgc-3′, CCR9 Reverse: 5′-ctgtggaagcagtggagtca, β-actin Forward: 5′-gctgtattcccctccatcgt -3′, β-actin Reverse: 5′-gccatgttcaatggggtact -5′. For nCounter™ analysis (Nanostring, Seattle, WA), code sets were generated by the GeneSelector algorithm [22]. 250 ng of colonic RNA were hybridized for 16 h, loaded onto the nCounter™ prep-station and quantified with the nCounter™ Digital Analyzer. nCounter data were normalized in two steps according to the manufacturer. We controlled for variations in the efficiency of processing with controls from the manufacturer. Data were normalized to Tomm7, Shfm1, Hprt1, Hsp90ab1 and actb as housekeeping genes.

### Cell culture and cytokine measurement

RPMI 1640 medium (GIBCO) supplemented with 10% FCS, 2 mmol/L L-glutamine, 100 U/ml penicillin-streptomycin, and 50 µmol/L 2-mercaptoethanol (Invitrogen) was used for all cultures. 10^6^ cells from WT and CCR9^−/−^ MLN harvested at d17 of DSS colitis were activated with immobilized anti-CD3 mAb (145-2C11; 0.5 µg/ml) and soluble anti-CD28 mAb (BD Biosciences). Supernatants were harvested at 72 h for cytokine measurement by BD Cytometric Bead Array (CBA) using a mouse Th1/Th2/Th17 kit according to the manufacturer's guidelines (BD Biosciences). Samples were analyzed on a FACS Canto II flow cytometer using FACS Diva Software (BD Biosciences) with FCAP Array Software (BD Biosciences).

### Enrichment of pDCs

pDCs were enriched by positive selection with PDCA-1 conjugated magnetic beads (Miltenyi Biotec, Auburn, CA) according to manufacturer. Purity was determined by flow cytometry using mAbs anti-MHC II, CD11c, PDCA-1 and B220 and Ly6C. Enrichment led to 90% purity.

### Statistical Analysis

Weight loss, Colon length, IBD Scoring and cellular distributions were compared using either Student t-test or ANOVA test. Differences with p<0.05 were considered significant. Statistical analysis was performed using Prism (Graph Pad Software, La Jolla, CA). Kaplan-Meier survival curves to DSS-mediated colitis were created using Prism (Graph Pad Software).

## Results

### CCR9 and CCL25 are expressed in the mucosa of the large intestine

**Figure 1:**
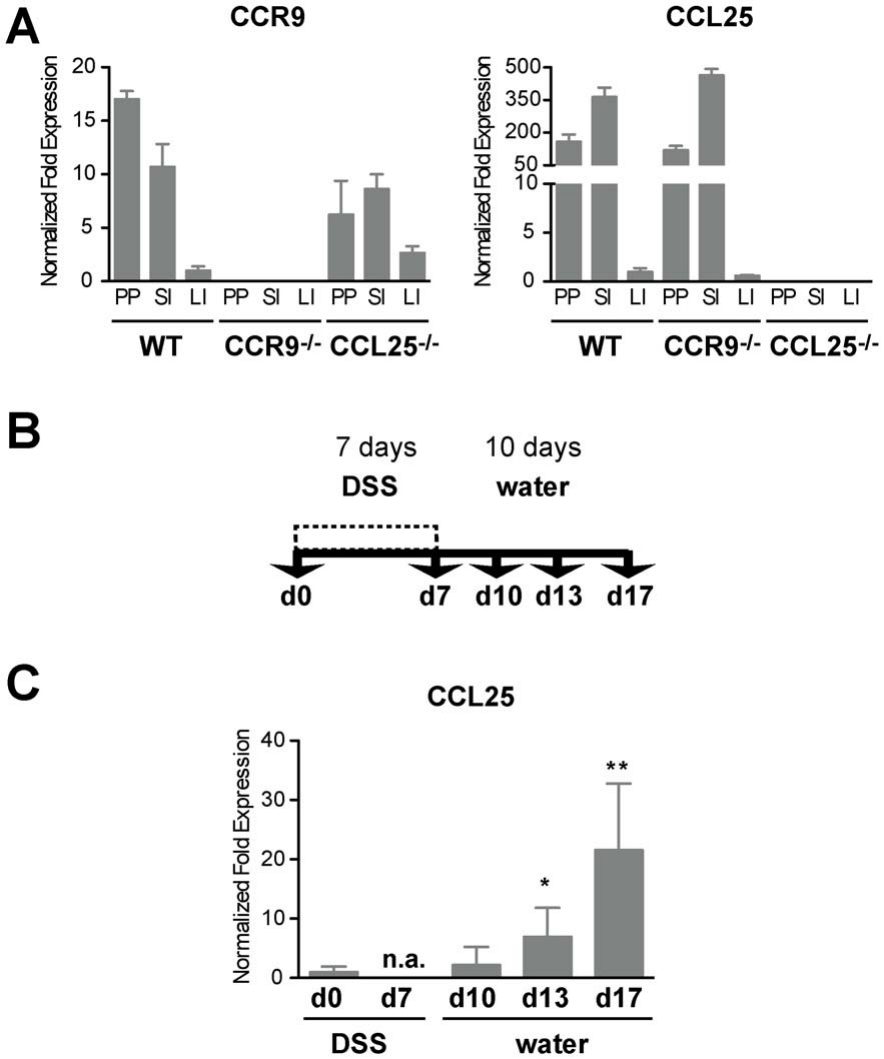
Expression of CCR9 and CCL25 in the large intestine. (**A**) Quantification of CCR9 and CCL25 mRNA levels from intestinal tissues of WT, CCR9^−/−^ and CCL25^−/−^ mice. Y-axis shows expression levels normalized to β-actin mRNA (mean +/− SD, n = 3). PP: Peyer's Patches, SI: Small intestine, LI: Large intestine. Data represent the mean +/− SD of 3 independent experiments, performed in triplicate (**B**) Acute DSS-mediated colitis. Induction phase: 2% DSS in drinking water for 7 days. Recovery: 10 days water. Mice were analyzed at days 0 (d0), d7, d10, d13 and d17. (**C**) CCL25 mRNA expression increases during the recovery phase of DSS-mediated colitis, (mean +/− SD; d0, n = 3; d10, n = 3; d13, n = 5; d17, n = 5; where n corresponds to a single experiments of 3 to 5 pooled intestinal samples).

In line with the literature [1], [2], [3], high levels of CCR9 and CCL25 transcripts were detected in Peyer's Patches (PPs) and in the small intestine (SI). We found low-level expression of CCR9 and CCL25 in the large intestine (LI) of healthy wild-type (WT) mice. Intestinal tissue from CCR9^−/−^ and CCL25^−/−^ animals was used as a specificity control (Figure 1*A*).

We next analyzed whether large intestinal inflammation affects expression levels of CCL25. Acute experimental colitis was induced by addition of DSS in the drinking water for seven days followed by a ten-day recovery period (Figure 1*B*). CCL25 mRNA levels did not change significantly until day 10 (d10). At d13 and at d17, CCL25 transcripts were significantly upregulated (6.9+/−4.8 and 21.5+/−11.2 fold induction, Figure 1*C*). Our data show that CCL25 expression increases during DSS colitis, suggesting a regulatory role of CCL25/CCR9 interactions during large intestinal inflammation.

### CCR9^−/−^ mice show increased susceptibility to acute DSS colitis

**Figure 2:**
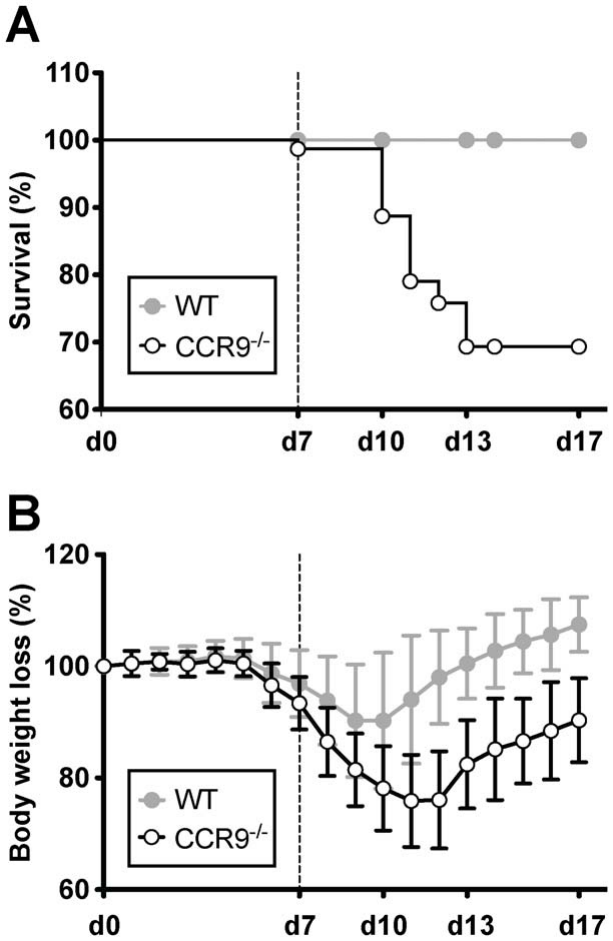
CCR9^−/−^ mice show increased mortality and delayed recovery during DSS-mediated colitis. (**A**) 30.65% mortality was observed in CCR9^−/−^ mice (open circles, n = 78). WT mice are shown in gray circles (n = 66). Data are representative of 17 pooled experiments. (**B**) CCR9^−/−^ mice showed a more pronounced body weight loss than WT. Body weight was monitored daily and is expressed as percent of weight at d0 (data represent the mean +/− SD of 17 pooled experiments of 3–5 mice per group). Open circles show CCR9^−/−^ mice, gray circles WT.

DSS colitis is dependent on the duration and dose of DSS administration as well as the genetic background of the animals [10], [23], [24]. We next compared the susceptibility of CCR9^−/−^ and WT mice to 3% DSS, as commonly used in C57Bl/6 mice. Surprisingly, administration of this DSS concentration induced a mortality of 100% in CCR9^−/−^ animals during the recovery phase (data not shown). We thus decreased the DSS concentration to 2% resulting in a reduction of mortality to 30.65% (Figure 2*A*). Both strains were susceptible to DSS colitis as evidenced by body weight loss (Figure 2*B*). Maximal weight loss occurred in WT mice by d10 and in CCR9^−/−^ mice by d12. CCR9^−/−^ mice lost significantly more weight than WT animals. In contrast to WT mice, CCR9^−/−^ animals were unable to regain their initial body weight during the water administration phase (Figure 2*B*). Like CCR9^−/−^ animals, CCL25^−/−^ mice were more susceptible to DSS colitis (Figure S1). Since similar results were obtained with the CCL25^−/−^ strain, these data show that the exacerbated colitis is a consequence of impaired CCL25/CCR9 interaction.

### Exacerbated IBD symptoms in CCR9^−/−^ animals

**Figure 3:**
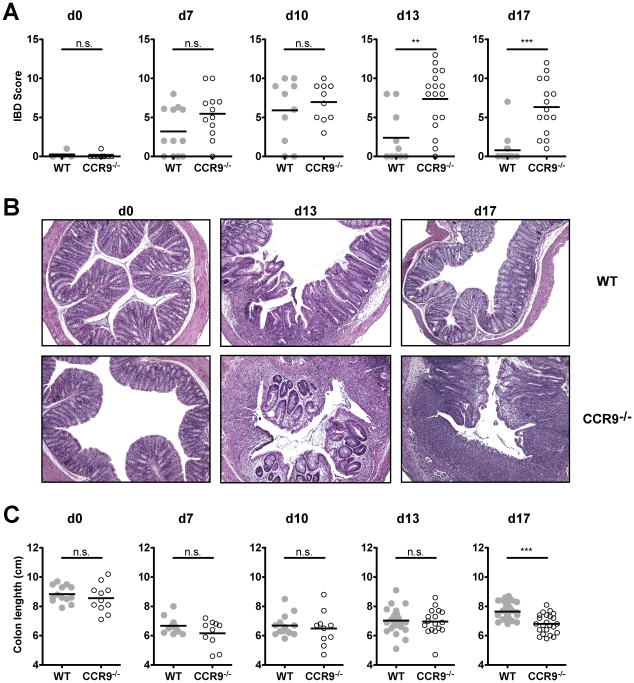
Exacerbation of DSS-mediated colitis in CCR9^−/−^ mice. (**A**) Higher IBD scores during the recovery phase in CCR9^−/−^ animals. WT mice are depicted in gray circles (n = 51), CCR9^−/−^ mice in open circles (n = 63). (**B**) Representative H&E sections from colons of WT and CCR9^−/−^ mice (d0, d13 and d17; 10×-magnification). CCR9^−/−^ intestinal mucosa displays exacerbated IBD symptoms. (**C**) Significant colon shortening in CCR9^−/−^ mice (open circles) at d17. Colon length was measured from d0 to d17. ***P<0.005, n.s. not significant.

We next analyzed how the increased susceptibility to DSS colitis affects IBD symptoms in CCR9^−/−^ animals. According to the weight loss data in WT animals, IBD scoring of H&E sections of the distal colon peaked at d10 and reversed during water administration by d13. In contrast, CCR9^−/−^ mice showed persistent intestinal inflammation until d17 (Figure 3*A* and Table S1). Intestinal inflammation in CCR9^−/−^ mice was characterized by an increase of cellular activity with granulocytic infiltration, expansion of the lamina propria and more pronounced basal lymphoplasmacytosis (Figure 3*B*). Epithelial hyperplasia, frequent crypt abscesses and mucosal ulceration also persisted until d17 (Figure 3*B*). As opposed to WT animals, CCR9^−/−^ mice still showed shortening of the colon at the end of the DSS cycle (Figure 3*C* and Table S2). CCL25 transcripts levels were not upregulated in CCR9^−/−^ large intestinal mucosa (Figure S2), probably due to the altered ratio of infiltrating cells to resident cells when compared to WT mucosa at the same time point (Figure S2). In summary, these experiments describe exacerbated IBD symptoms in the absence of CCR9 expression.

### The gut-associated lymphoid tissue and the intestinal mucosa of CCR9^−/−^ mice show multiple signs of exacerbated inflammation after DSS colitis

**Figure 4:**
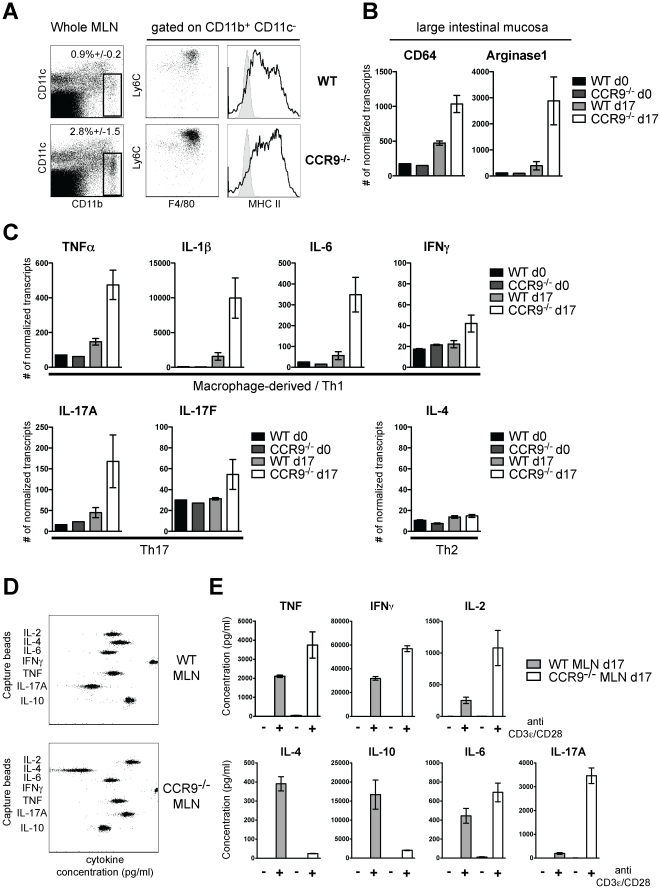
The gastrointestinal tract of CCR9^−/−^ animals shows multiple signs of exacerbated tissue inflammation. (**A**) Accumulation of inflammatory macrophages. Flow cytometry analysis of MLNs of WT and CCR9^−/−^ animals. Frequencies of inflammatory macrophages were determined in MLN suspensions with anti-CD11c, CD11b, F4/80, Ly6C and MHC-II (in black) overlaid on isotype control mAbs (in gray). Data represent the mean (+/− SD) of 3 independent experiments of 3–5 pooled mice per genotype. (**B and C**) mRNA profiling of large intestinal tissues. Total RNA was prepared from the colon of WT and CCR9^−/−^ mice at d0 and d17 and was analyzed with the nCounter™ system (mean +/− SD of 3–5 animals per group of 2 independent experiments). (**B**) Macrophage accumulation is reflected by increased mRNA levels of CD64 and Arginase1. (**C**) Comparison of tissue immunophenotype of the large intestinal mucosa before and after DSS-mediated colitis in WT and CCR9^−/−^ animals. (**D and E**) T cell re-stimulation assay for cytokine production. (**D**) Representative cytometry bead array assay for Th1/Th2/Th17 cytokines. (**E**) Production of TNFα, IFNγ, IL-2, IL-6 and IL-17A is upregulated while production of IL-10 and IL-4 is down-regulated in T cells from CCR9^−/−^ animals. Quantification of cytokine production from WT (gray bars) and CCR9^−/−^ cells (open bars). Data represent one experiment out of 3 independent experiments, performed in triplicate (mean +/− SD) of 3 pooled WT MLN and 3 pooled CCR9^−/−^ MLN cell suspensions reactivated in vitro with anti-CD3ε/anti-CD28 mAbs for 72 h.

As recruitment and activation of intestinal MΦ has been described in human ulcerative colitis and murine DSS colitis [10], [17], we analyzed the numbers and phenotype of MΦ in gut-associated lymphoid tissue and intestinal mucosa. The cellularity of lymphoid organs during DSS colitis was similar in WT and CCR9^−/−^ animals (Figure S3). At d17, however, a 3-fold increase of MΦ was detected in MLNs of CCR9^−/−^ animals compared to WT mice. MΦ in both strains expressed the surface markers F4/80 and Ly6C, suggesting an inflammatory phenotype [15] (Figure 4*A*). CCR9^−/−^ MΦ expressed higher levels of MHC II than WT cells, indicating that these MΦ are more activated [25].

We next compared mRNA expression patterns of the large intestinal tissue of WT and CCR9^−/−^ animals at steady state and at d17 using nCounter™ mRNA analysis. This digital methodology allows for a direct analysis of mRNA copy numbers without amplification steps [22]. In line with our flow cytometry analysis, the increase in inflammatory MΦ at d17 was reflected by increased expression of CD64 and Arginase1 (Figure 4*B*). As a reflection of recent tissue inflammation, mRNA levels of TNFα, IL-1β and IL-6 were increased at d17 (Figure 4*C*). CCR9^−/−^ animals consistently showed increased levels of all transcripts when compared to WT (Figures 4*B* and 4*C*). Exacerbated IBD in CCR9^−/−^ mice further correlated with upregulation of IFNγ, IL-17A and IL-17F. No induction of IL-4 mRNA was observed (Figure 4*C*).

We next studied cytokine production of T cells isolated from MLNs at d17 after stimulation with anti-CD3ε/CD28 mAbs (Figure 4*D* and 4*E*). T cells from CCR9^−/−^ animals produced higher levels of TNFα, IFNγ, IL-2, IL-6 and IL-17A than WT cells. Production of IL-4 and IL-10 was significantly down-regulated in CCR9^−/−^ mice. In summary, this set of data describes an exacerbated inflammatory immune response of the Th1/Th17 immunophenotype with higher numbers of tissue macrophages in the large intestinal mucosa and gut-associated lymphoid tissues of CCR9^−/−^ mice.

### Exacerbated DSS colitis correlates with the accumulation of activated plasmacytoid DCs in MLNs

DCs are gatekeepers for the induction of adaptive immune responses and different DC subpopulations are also considered key regulators of inflammatory immune responses [14]. In particular, expression of CCR9 on plasmacytoid DCs (pDCs) defines a tolerogenic subpopulation [26]. We therefore compared the distribution of DC subsets in CCR9^−/−^ and WT animals at steady state and during DSS colitis.

**Figure 5:**
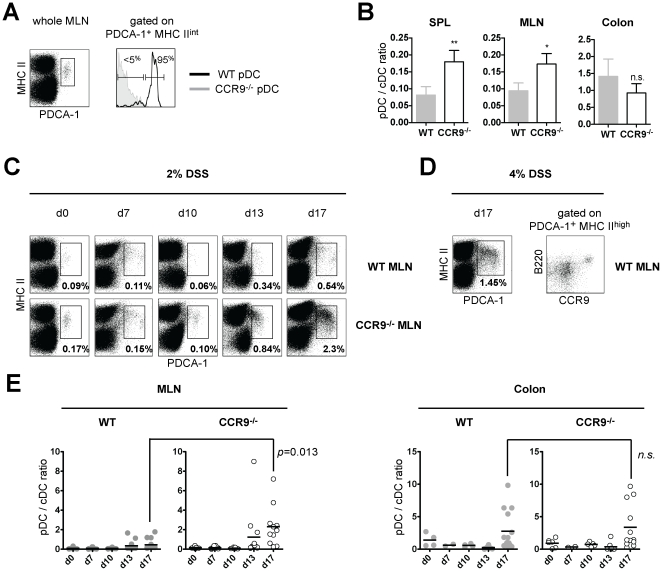
CCR9^−/−^ mice show altered ratios of dendritic cell subpopulations at steady state and during DSS-mediated colitis. (**A**) WT MLNs contain CCR9^+^ and CCR9^−^ pDCs gated as MHC II^int^ and PDCA-1^high^ (left dot plot). CCR9 expression in WT pDCs (black line, right histogram) is overlaid on CCR9^−/−^ pDCs (in gray, right histogram) (**B**) Steady state distribution of pDCs and cDCs is altered in SPL and MLN of CCR9^−/−^ mice. No significant difference between strains is observed in the colon. cDCs were defined as CD11c^high^ and MHC II^high^ cells by flow cytometry. Ratio of subpopulations is expressed on the y-axis as pDC/cDC cells. Data represent the mean +/− SD of 4 independent experiments of 3–5 pooled mice per group. (**C**) Accumulation of pDCs in MLNs of CCR9^−/−^ animals. Percentages of pDCs in WT and CCR9^−/−^ MLN are depicted in bold from d0 to d17 of DSS colitis. (**D**) Accumulation of pDCs correlates with the level of tissue damage. Increase of DSS concentration to 4% augments the number of CCR9^low/−^ B220^+^ PDCA-1^+^ MHC II^int/high^ cells in MLNs of WT animals. (**E**) DSS-mediated tissue inflammation alters pDC/cDC ratios in MLN of CCR9^−/−^ animals. Data points correspond to individual experiments corresponding to 17 DSS colitis experiments harvested at different time point using 3–5 pooled mice per genotype.

We first confirmed that a subset of pDCs from MLNs and spleen of WT animals expresses high levels of CCR9 [26], [27] (Figure 5*A* and data not shown). Then, we analyzed distribution of DC subpopulations in CCR9^−/−^ and WT animals by assessing the ratio between pDCs and conventional DCs (cDCs). According to the literature [15], cDCs were gated based on expression of high levels of CD11c and MHC II pDCs were defined as MHC II^low^, CD11c^low^ and PDCA-1^+^ cells [28]. The relative distribution of cDCs subsets based on expression of CD8α, CD11b and CD103 did not differ in lymphoid organs or large intestinal tissues at steady state between the two strains (data not shown). In line with the literature, our results showed an increase in the pDC/cDC ratio in SPL and MLN due to an accumulation of pDCs in CCR9^−/−^ mice (Figure 5*B*). The pDC/cDC ratio in the colon of both strains was comparable.

We next analyzed the effects of DSS colitis on the distribution of pDCs in MLNs and observed a biphasic pattern. At d10, a drop in pDC numbers was detected in WT and CCR9^−/−^ mice. Then, we observed an accumulation of pDCs, which was significantly more pronounced in CCR9^−/−^ when compared to WT animals (2-fold at d13, 4-fold at d17, Figure 5*C*). The enhanced accumulation of pDCs observed in CCR9^−/−^ animals significantly shifted the pDC/cDC ratio in MLNs at d13 and d17. The relative distribution of cDCs and their subsets during DSS colitis was comparable in both strains (Figure S4 and S5). The pDC/cDC ratio in the colon (Figure 5E) and in the SPL (Figure S6) was not affected.

Since the severity of colitis symptoms in mice is partly dependent on the DSS dose [10], [23], we hypothesized that a higher dose of DSS might induce a comparable accumulation of pDCs in MLNs of WT animals. Administration of 4% DSS indeed induced accumulation of pDCs when compared to 2% DSS (Figure 5*C* and 5*D*). We detected a pDC subset that expresses CCR9. The majority of accumulating pDCs, however, were lacking CCR9 expression (Figure 5*D*), implying that these pDCs have an inflammatory rather than a tolerizing immune phenotype [26].

**Figure 6:**
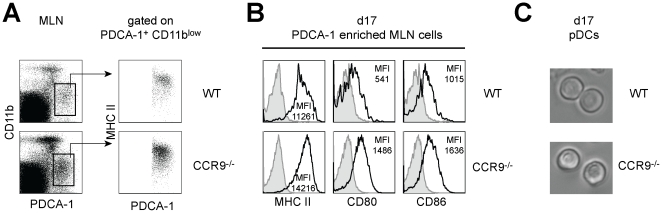
pDCs from CCR9^−/−^ MLNs are more activated than WT pDCs. (**A**) pDCs of WT and CCR9^−/−^ mice express low levels of CD11b in contrast to inflammatory macrophages. pDCs were defined as CD11b^low^ MHC II^int/high^ PDCA-1^+^. (**B**) CCR9^−/−^ pDCs show signs of phenotypic maturation. Maturation levels were assessed by comparing expression levels of MHC II and costimulatory molecules CD80 and CD86. (**C**) Morphology of pDCs isolated from WT and CCR9^−/−^ animals.

We compared maturation/activation levels of the accumulating pDCs by comparing MHC II, CD80 and CD86 expression levels. Low expression levels of CD11b on WT and CCR9^−/−^ pDCs (Figure 6*A*, left) confirmed that these cells were different from the activated macrophages described in Figure 4. By gating on the CD11b^low^ PDCA-1^+^ population, we confirmed that the pDCs were MHC II^+^ (Figure 6*A*, right) and CD11c^low^ and Ly6C^high^ (data not shown). Comparative analysis of expression levels of MHC II, CD80 and CD86 showed that these surface markers were upregulated in pDCs from CCR9^−/−^ animals (Figure 6*B*). Despite the different activation level, pDCs isolated from MLNs of WT and CCR9^−/−^ animals at d17 showed the same plasmacytoid morphology (Figure 6*C*). In summary, these data show that activated pDCs accumulate in the gut-associated lymphoid tissue of CCR9^−/−^ animals.

## Discussion

The results presented in this study identify a novel role of CCL25/CCR9 interactions in the pathogenesis of a well-established acute colitis model mediated by oral administration of DSS. In our study, DSS specifically targets the large intestine, but not the small intestine, and we show that CCL25 expression is upregulated after acute large intestinal inflammation. When studying CCR9^−/−^ mice with the DSS model, we found that the large intestinal mucosa displays characteristics of enhanced tissue inflammation resulting in exacerbated IBD symptoms and a delay in recovery from tissue injury. Thus, our findings are of high relevance for clinical care of human IBD as they point to potential detrimental effects of CCR9-blocking therapy for patients suffering from inflammation in the large intestine, as commonly observed in ulcerative colitis. This suggests that blocking CCL25/CCR9 interactions in ulcerative colitis patients may lead to an inefficiency in resolving intestinal inflammation or, at worst case, even exacerbated IBD symptoms. An oral CCR9 antagonist (CCX282-B) is currently used in a Phase II clinical trial for the treatment of Crohn's disease and has shown efficacy in the treatment of patients with moderate to severe small bowel Crohn's disease [8]. This compound has shown potent inhibition of CCL25-mediated chemotaxis of CCR9-expressing cells [29]. However, contradicting results have emerged in the role of CCL25/CCR9 interactions in the experimental small intestinal inflammation of the TNF^Δare^ mouse model that mimics human Crohn's Disease. Walters et al. showed that the orally bioavailable CCR9 antagonist CCX282-B can ameliorate, in one single experiment, the severity of small intestinal chronic inflammation of TNF^Δare^ mice [29]. Apostolaki et al. demonstrated that the development of intestinal inflammation in the TNF^Δare^ mouse model is critically dependent on β7 integrin-mediated T-lymphocyte recruitment, while the function of the CCL25/CCR9 axis in T-cell specific mucosal recruitment appears dispensable in this model [30]. The fact that Walters et al. did not provide the CCR9 antagonist orally but subcutaneously may explain such discrepancy. Oral administration of CCR9 antagonists should thus be evaluated in animal models of large intestinal inflammation.

In the SAMP1/Yit chronic ileitis mouse model that shares similarities with the TNF^Δare^ mouse model, Matsuzaki et al. showed that antibodies against the α4β7-integrin ligand MAdCAM-1 significantly inhibited adhesion of T lymphocytes to microvessels of the terminal ileum of SAMP1/Yit mice, and led to a strong chronic ileitis suppressive effect [31]. Although antibody blockade of CCL25/CCR9 interactions ameliorated early, but not late, SAMP1/Yit chronic murine ileitis [32], it would be very interesting to test whether the CCR9 antagonist CCX282-B and/or CCR9-deficiency in the SAMP1/Yit ileitis model can ameliorate small intestinal chronic inflammation as well. The fact that these studies were focused on small intestinal but not large intestinal inflammation, made CCL25 and CCR9 attractive candidates for the treatment of Crohn's disease but not ulcerative colitis. However, prior to this study, no work has been presented on CCL25/CCR9 interactions in an experimental acute colitis mouse model mediated by DSS exposure.

Our data on the gastrointestinal expression pattern of CCL25 support the findings of Stenstad et al. showing expression of this chemokine in the colon [33]. To date, only low expression levels at steady state were described. We show here that inflammation increases CCL25 expression in the large intestinal mucosa. It has been described that the small intestinal mucosa reacts to injection of the inflammatory cytokine TNFα with rapid upregulation of CCL25 [34]. Our data suggest that the colonic mucosa reacts similarly to inflammation, presumably to attract CCR9-positive leukocytes to participate in the mucosal immune response.

The most compelling argument for a physiologic role of CCL25/CCR9 interactions during an inflammatory response in the colon is derived from the increased susceptibility to colitis and the high mortality rate observed in CCR9^−/−^ animals. Increased IBD scores, more pronounced weight loss, a delay in recovery from acute inflammation and the inability to regain initial body weight during the experimental time period support the critical role for CCL25/CCR9 dependent trafficking events during the acute and recovery phase of DSS colitis. The altered microenvironment of the large intestine appears to favor CCL25/CCR9-independent migration of pro-inflammatory leukocytes in CCR9^−/−^ animals. This situation is highly relevant for our understanding of the therapeutic effects of CCR9-antagonists as this type of medication *could* result in a comparable scenario in human IBD patients.

To date, targeting of chemokine receptors such as CCR2, CCR5, CCR6, CXCR3, CXCR4 and CX3CR1 by gene disruption or antagonistic peptides has been found to protect animals from DSS colitis [35], [36], [37], [38], [39]. The mechanism commonly proposed is that interference with chemokine receptor function is beneficial, because these receptors are potentially pro-inflammatory. In contrast, we show that CCR9^−/−^ and CCL25^−/−^ animals respond with exacerbated IBD symptoms. To our knowledge, this is the first report to show that interference with a chemokine/receptor pair aggravates IBD symptoms. As a mechanism, we propose that CCL25/CCR9 interactions are critical components of an anti-inflammatory tissue response in the colon.

The interpretation of our results as a consequence of an impaired anti-inflammatory response in CCR9^−/−^ animal is supported by our data in two ways: the pronounced accumulation of activated inflammatory macrophages and the increased expression of the pro-inflammatory cytokines IL-1β, IL-6, and TNF-α in the large intestinal mucosa. Expression of the latter cytokines is not only described for animal models of colitis [40], [41] but also for human IBD [42], [43], underlining the relevance of the murine model for human disease. Unlike other models of murine colitis [44], our model shows that the absence of CCR9 does not change the immune profile of the inflammatory response. Only higher levels of Th1- and Th17-type inflammatory cytokines, but no Th2-type immune responses, were detected. Additionally, high levels of IL-17A and IL-17F were detected as part of the aggravated immunophenotype of CCR9^−/−^ mice both at the mRNA level and the protein level. As IL-17 is considerably increased in chronic IBD patients [45], it is conceivable that the CCL25/CCR9 interactions are playing a physiological role during the development of the chronic disease symptoms as well. Melgar *et al.* showed that a single cycle of high-dose DSS can induce chronic inflammation over time in wild type C57Bl/6 mice [24]. Along this line, we have preliminary evidence that a single cycle of low-dose DSS is sufficient in CCR9^−/−^ C57Bl/6 animals to induce chronic IBD (data not shown). Further investigation will determine whether CCL25/CCR9 interactions play a role in DSS chronic colitis.

The delayed recovery from acute colitis is further characterized by an accumulation of pDCs and a change of the pDC/cDC ratio in the gut-associated lymphoid tissue in CCR9^−/−^ mice. pDCs have been described as immune suppressive APCs [46] that can induce systemic tolerance to T cell-mediated delayed-type hypersensitivity after oral sensitization [47]. pDCs are also discussed as regulators of DSS colitis [48]. However, Hadeiba et al. described a significant difference in the ability of pDCs subsets to promote tolerance [26]. In fact, CCR9 expression delineates a tolerogenic pDC subset while CCR9-negative pDCs were unable to prevent T cell activation/proliferation. Our results also suggest the concerted action of two functionally distinct pDCs subsets: a CCR9-positive tolerogenic pDC subset that regulates the inflammatory response during and after DSS colitis and a CCR9-negative predominantly inflammatory pDC subset. When regulatory pDCs lose CCR9 expression due to genetic manipulation, they also lose their ability to migrate in response to CCL25 upregulation in the colon and become trapped in the MLN. Alternatively, CCR9 expression by itself could influence the tolerogenic potential of these pDCs. One might even argue that the subset of CCR9-positive regulatory pDCs is underrated in WT animals, as these cells are likely to escape detection by flow cytometry once they have reached large intestinal LP. These cells likely down-regulate their surface chemokine receptors in response to CCL25. The CCR9-negative pDCs appear inflammatory in our model as demonstrated by the correlation of exacerbated IBD symptoms with the expansion of this pDC subset. In CCR9^−/−^ mice, the altered microenvironment might also favor CCL25/CCR9-independent tissue homing of inflammatory pDCs. Additionally, the impaired balance of DCs subpopulations may contribute to the enhanced activation of the immune system and the failure to recover from tissue injury in the absence of physiological CCL25/CCR9 interactions. It now will be important to study if the impaired ratio of inflammatory and regulatory pDCs, the expanded macrophage population or both cell types regulate the exacerbated IBD symptoms in CCR9^−/−^ animals. The question whether this phenotype is actually exacerbated IBD or rather an impaired ability to recover from acute large intestinal inflammation also requires further analysis.

Prevention of the CD45RB^high^ CD4+ T cell transfer colitis model in immuno-compromised animals can be achieved by cotransferring Regulatory T cells (Tregs) [49]. Given the fact that CCR9-expressing pDCs are inducers of T regulatory cell functions, further examination of Tregs distribution and function would determine whether Tregs play a role in DSS colitis recovery and whether or not it is CCL25/CCR9 dependent.

In summary, our study shows that impairment of CCL25/CCR9-interaction has profound negative effects on the regulation of the local inflammatory immune response. While a phase II clinical study with a CCR9-inhibitor suggests a therapeutic benefit for Crohn's disease patients [8], our results strongly suggest that this form of therapeutic intervention could have detrimental effects when offered to patients that suffer from ulcerative large intestinal-specific inflammation.

## Supporting Information

Figure S1 **CCL25^−/−^ mice show increased susceptibility to DSS colitis comparable to CCR9^−/−^ animals.** (**A**) A mortality rate of 38% was observed in CCL25^−/−^ (blue circles) mice exposed to DSS for 7 days and 10 days of water when compared to control CCL25^−/−^ mice exposed to water only (black circles). Data represent survival of 5 mice per group in 5 independent experiments; P value = 0.0166 (Log-rank Test). (**B**) Delayed recovery in CCL25^−/−^ mice is evidenced by significant weight loss and inability to recover to initial body weight. Body weight was monitored daily and is expressed as percent of body weight measured at d0. Data are displayed as the mean +/− SD of the mean of 5 independent experiments, each with 5 mice per group. Blue circles show CCL25^−/−^ mice exposed to DSS colitis, black circles CCL25 control mice.
(TIF)

Figure S2 **CCL25 mRNA expression increases during the recovery phase of DSS-mediated colitis in WT intestinal mucosa but not CCR9^−/−^ large intestinal mucosa.** Y-axis shows expression CCL25 levels normalized to β-actin mRNA. Data represent the mean +/− SD; d10, n = 3; d13, n = 5; d17, n = 5; where n corresponds to the number of experiments of 3 to 5 pooled large intestinal samples.
(TIF)

Figure S3 **Cellularity in SPL and MLN of WT and CCR9^−/−^ animals during DSS colitis is comparable.** Collagenase digestion of SPL and MLN was performed at indicated time points. Cell suspensions were counted with a Casy® automatic cell counter. Each data point represents a single mouse, WT mice are depicted in gray circles and CCR9^−/−^ mice are depicted in open circles. Horizontal black bars represent the mean of SPL and MLN cell numbers from d0 to d17. SPL: spleen, MLN: mesenteric lymph nodes.
(TIF)

Figure S4 **Distribution of pDCs and cDCs in WT and CCR9^−/−^ mice during DSS colitis.** Cell suspensions were prepared from WT and CCR9^−/−^ SPL, MLN and colonic mucosa and were analyzed by flow cytometry. Frequencies of pDCs (PDCA-1^+^ MHC II^int/high^) and cDCs (CD11c^high^ MHC II^high^) were determined and are graphed as percentages of total leukocytes. Each data point represents a single animal. WT mice are depicted as gray circles and CCR9^−/−^ mice as open circles. Horizontal black bars represent the mean of pDCs and cDCs frequencies determined by flow cytometry from d0 to d17.
(TIF)

Figure S5 **Flow cytometry analysis of cDCs subsets in WT and CCR9^−/−^ animals at d17 of DSS colitis.** Cell suspensions from SPL, MLN and colonic mucosa were analyzed by flow cytometry. Frequencies of cDCs subsets (CD11c^high^ MHC II^high^) were determined and subsets were further analyzed based on expression of CD11b and CD103. Percentages of cDCs subsets are expressed as percentage of total cDCs and numbers are shown in red in each graph. Dot plots are representative of 5 independent experiments. We did not observe any statistically significant differences in the frequencies of all cDC subsets analyzed.
(TIF)

Figure S6 **Ratio of DC subsets in the spleen of CCR9^−/−^ animals compares to WT spleens during DSS colitis.** Quantification of pDC/cDC ratios at d0, d7, d10, d13 and d17 in WT (gray circles, left graph) and CCR9^−/−^ mice (open circles, right graph) by flow cytometry using a lineage negative staining to remove any CD3^+^, CD19^+^ and CD11b^high^ cells. Each data point corresponds to one experiment using 3–5 pooled animals. Horizontal black bars represent the mean of pDC/cDC ratios from d0 to d17.
(TIF)

Table S1 **IBD scoring.** Values represent the mean ± SEM of IBD scores depicted in Figure 3a.
(DOC)

Table S2 **Colon Length.** Values represent the mean ± SEM of colon length depicted in Figure 3c.
(DOC)
