# Supplementary figures and images for: CCL25/CCR9 Interactions Regulate Large Intestinal Inflammation in a Murine Model of Acute Colitis
Source: PLoS One. 2011 Jan 25;6(1):e16442. doi: 10.1371/journal.pone.0016442 (PMC3026821; doi:10.1371/journal.pone.0016442)

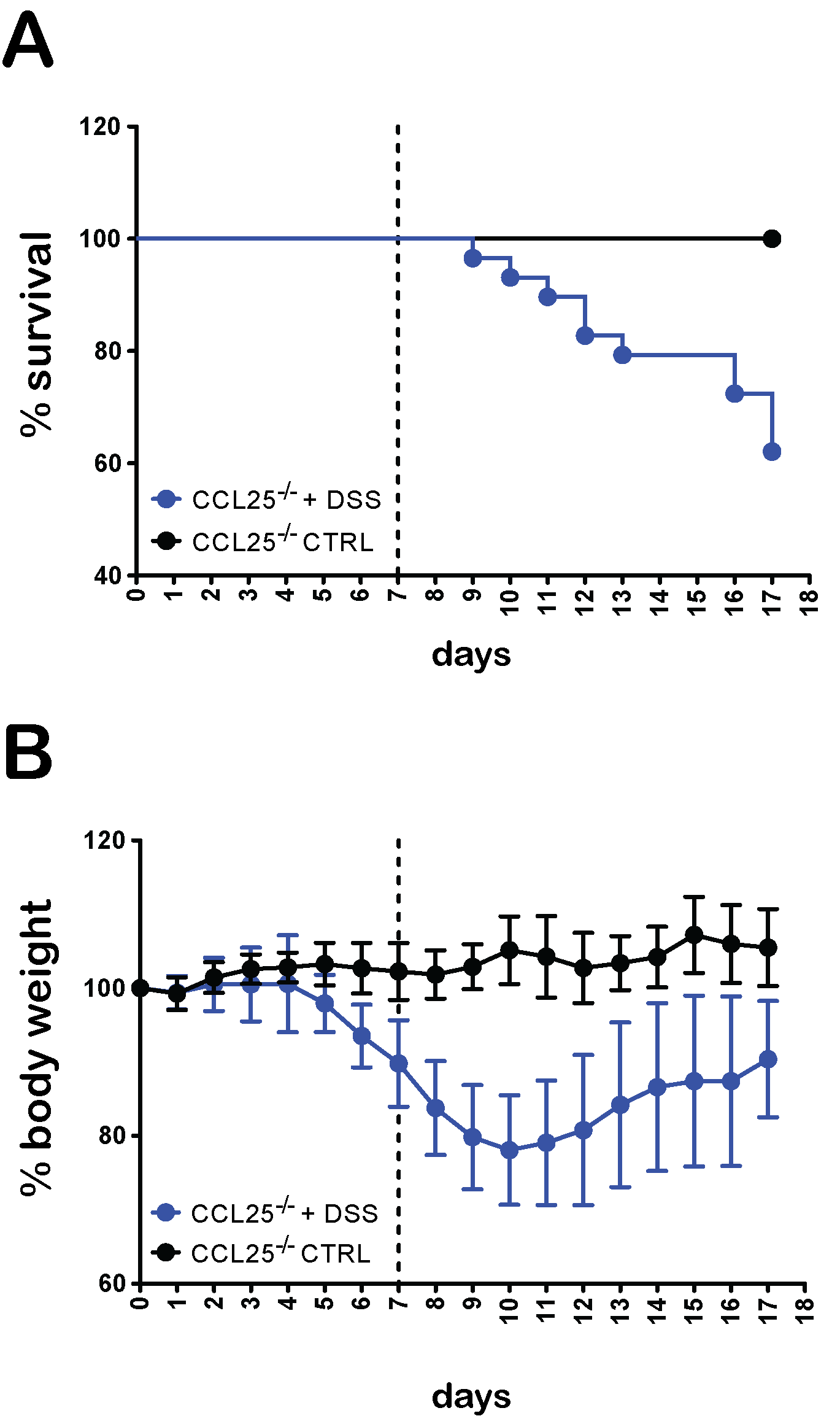

Supplement: Figure S1 — CCL25−/− mice show increased susceptibility to DSS colitis comparable to CCR9−/− animals. (A) A mortality rate of 38% was observed in CCL25−/− (blue circles) mice exposed to DSS for 7 days and 10 days of water when compared to control CCL25−/− mice exposed to water only (black circles). Data represent survival of 5 mice per group in 5 independent experiments; P value = 0.0166 (Log-rank Test). (B) Delayed recovery in CCL25−/− mice is evidenced by significant weight loss and inability to recover to initial body weight. Body weight was monitored daily and is expressed as percent of body weight measured at d0. Data are displayed as the mean +/− SD of the mean of 5 independent experiments, each with 5 mice per group. Blue circles show CCL25−/− mice exposed to DSS colitis, black circles CCL25 control mice. (TIF) [file pone.0016442.s001.tif]

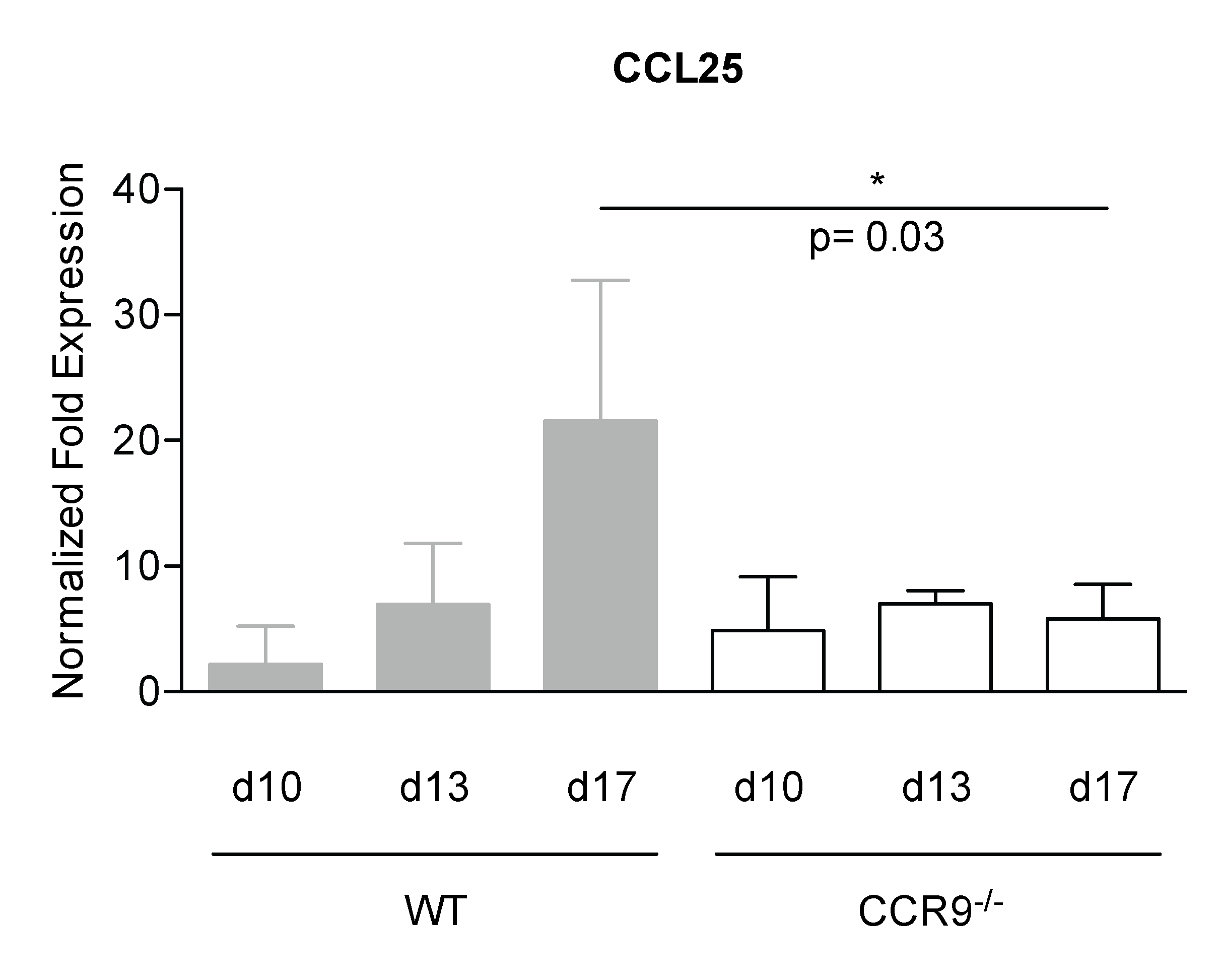

Supplement: Figure S2 — CCL25 mRNA expression increases during the recovery phase of DSS-mediated colitis in WT intestinal mucosa but not CCR9−/− large intestinal mucosa. Y-axis shows expression CCL25 levels normalized to β-actin mRNA. Data represent the mean +/− SD; d10, n = 3; d13, n = 5; d17, n = 5; where n corresponds to the number of experiments of 3 to 5 pooled large intestinal samples. (TIF) [file pone.0016442.s002.tif]

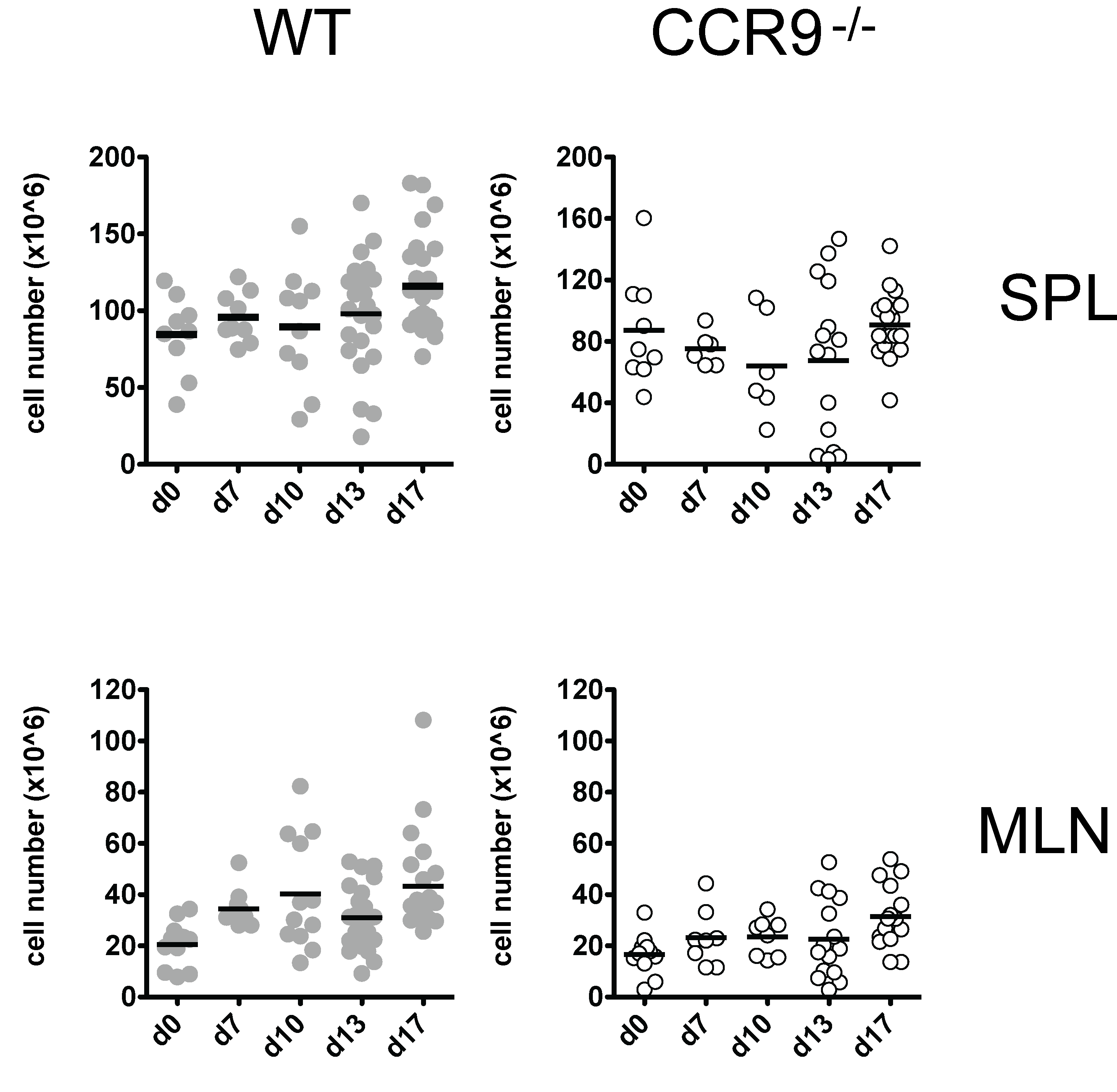

Supplement: Figure S3 — Cellularity in SPL and MLN of WT and CCR9−/− animals during DSS colitis is comparable. Collagenase digestion of SPL and MLN was performed at indicated time points. Cell suspensions were counted with a Casy® automatic cell counter. Each data point represents a single mouse, WT mice are depicted in gray circles and CCR9−/− mice are depicted in open circles. Horizontal black bars represent the mean of SPL and MLN cell numbers from d0 to d17. SPL: spleen, MLN: mesenteric lymph nodes. (TIF) [file pone.0016442.s003.tif]

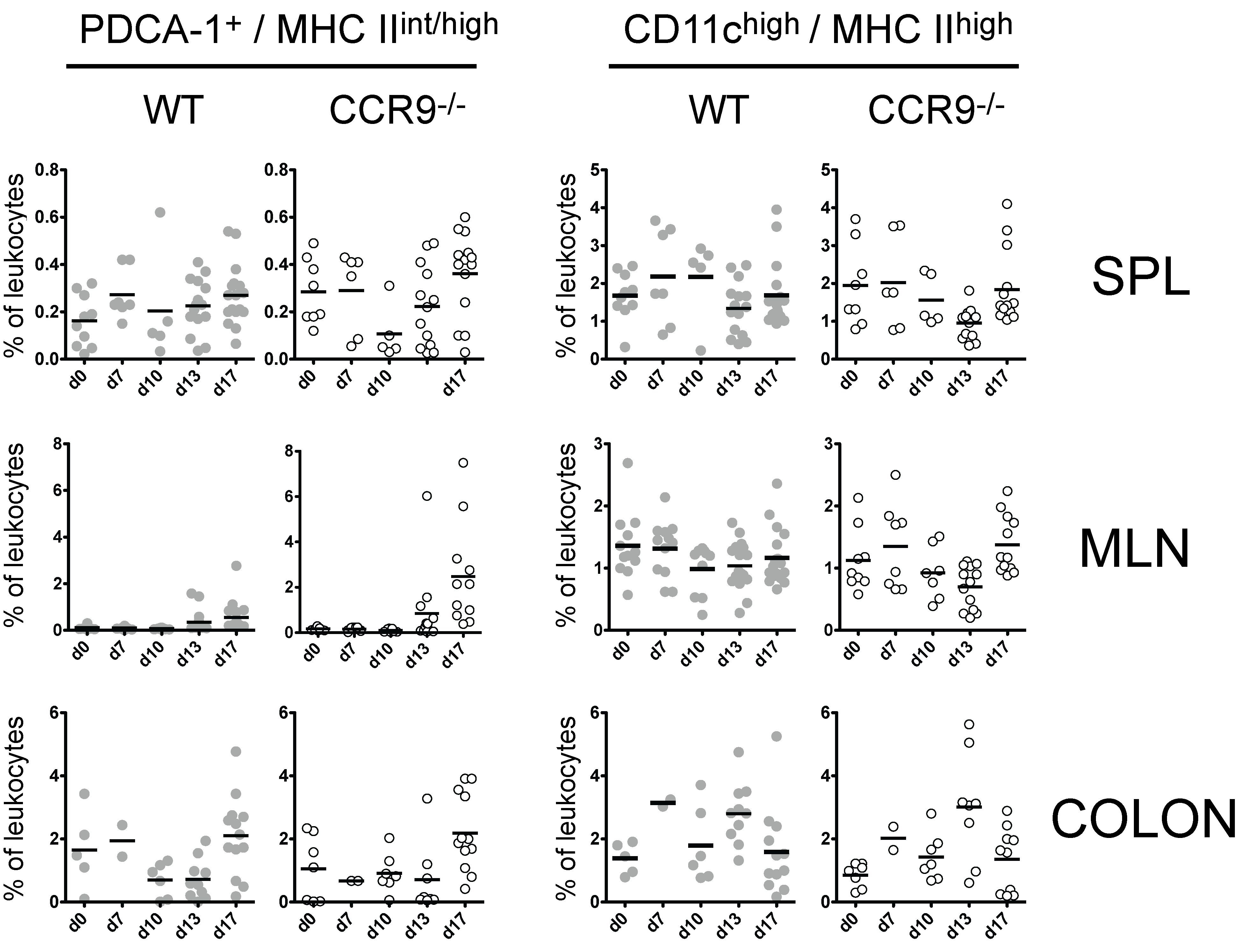

Supplement: Figure S4 — Distribution of pDCs and cDCs in WT and CCR9−/− mice during DSS colitis. Cell suspensions were prepared from WT and CCR9−/− SPL, MLN and colonic mucosa and were analyzed by flow cytometry. Frequencies of pDCs (PDCA-1+ MHC IIint/high) and cDCs (CD11chigh MHC IIhigh) were determined and are graphed as percentages of total leukocytes. Each data point represents a single animal. WT mice are depicted as gray circles and CCR9−/− mice as open circles. Horizontal black bars represent the mean of pDCs and cDCs frequencies determined by flow cytometry from d0 to d17. (TIF) [file pone.0016442.s004.tif]

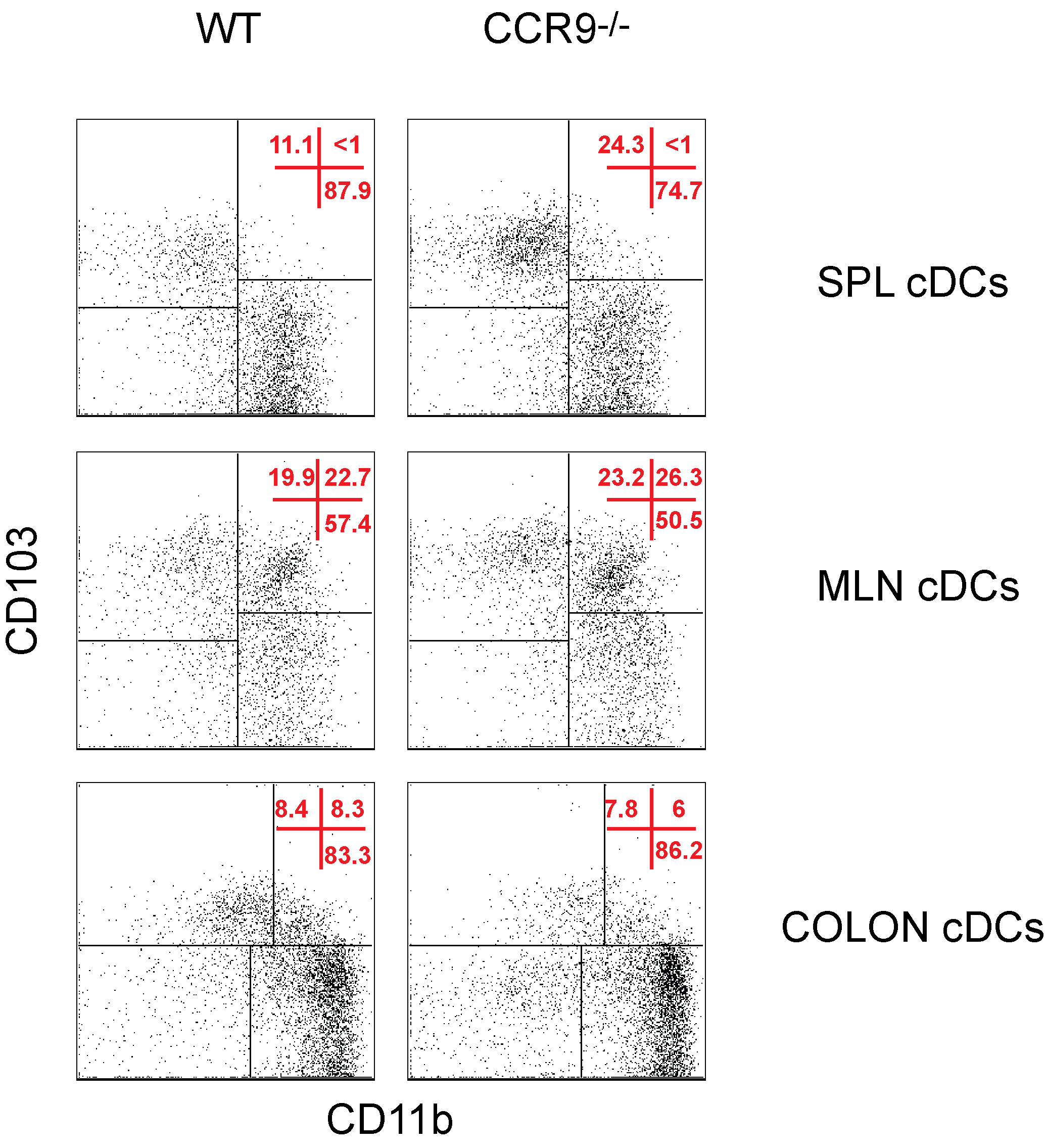

Supplement: Figure S5 — Flow cytometry analysis of cDCs subsets in WT and CCR9−/− animals at d17 of DSS colitis. Cell suspensions from SPL, MLN and colonic mucosa were analyzed by flow cytometry. Frequencies of cDCs subsets (CD11chigh MHC IIhigh) were determined and subsets were further analyzed based on expression of CD11b and CD103. Percentages of cDCs subsets are expressed as percentage of total cDCs and numbers are shown in red in each graph. Dot plots are representative of 5 independent experiments. We did not observe any statistically significant differences in the frequencies of all cDC subsets analyzed. (TIF) [file pone.0016442.s005.tif]

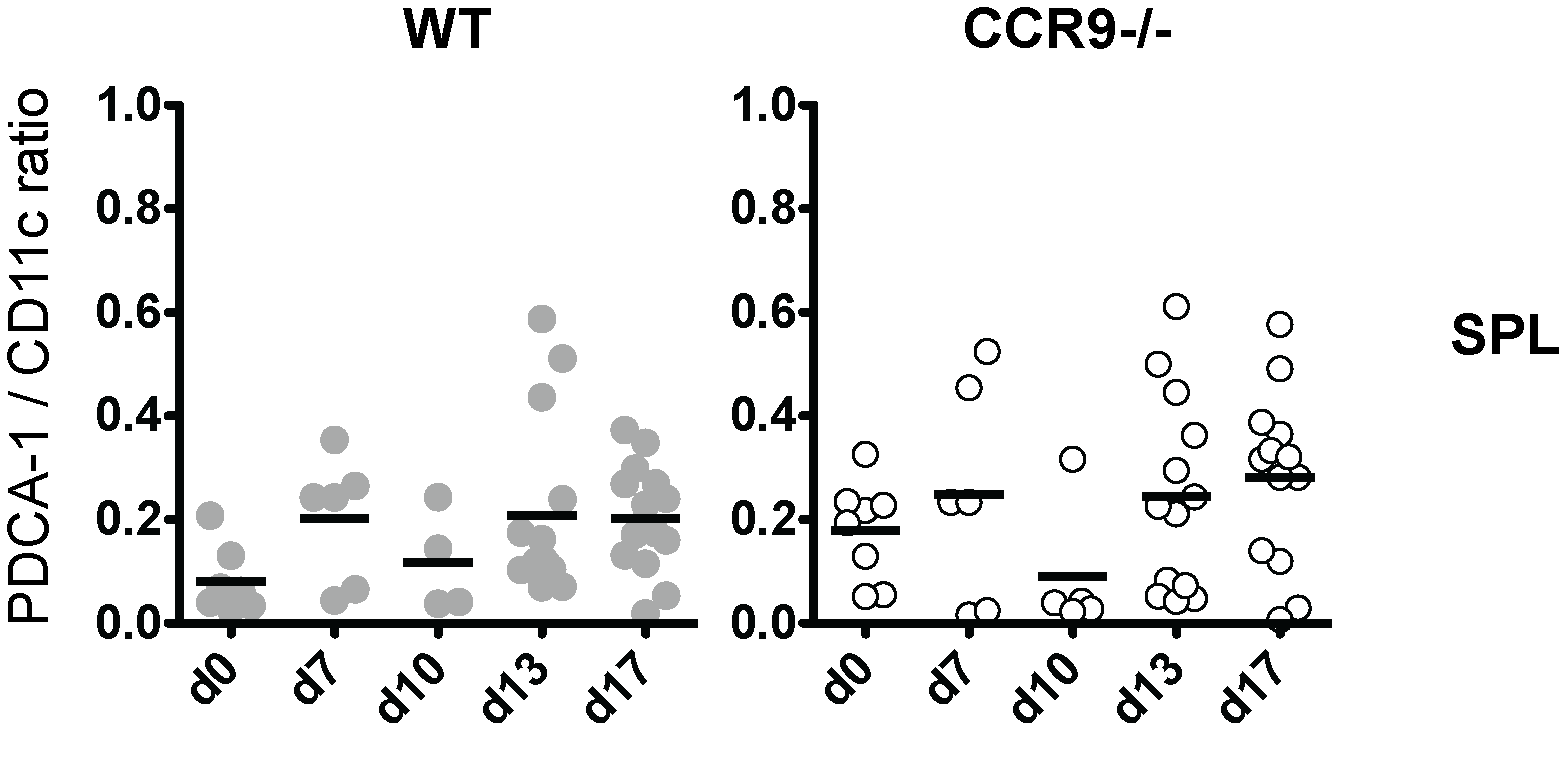

Supplement: Figure S6 — Ratio of DC subsets in the spleen of CCR9−/− animals compares to WT spleens during DSS colitis. Quantification of pDC/cDC ratios at d0, d7, d10, d13 and d17 in WT (gray circles, left graph) and CCR9−/− mice (open circles, right graph) by flow cytometry using a lineage negative staining to remove any CD3+, CD19+ and CD11bhigh cells. Each data point corresponds to one experiment using 3–5 pooled animals. Horizontal black bars represent the mean of pDC/cDC ratios from d0 to d17. (TIF) [file pone.0016442.s006.tif]
