# Supplementary material for: CCL25/CCR9 Interactions Regulate Large Intestinal Inflammation in a Murine Model of Acute Colitis
Source: PLoS One. 2011 Jan 25;6(1):e16442. doi: 10.1371/journal.pone.0016442 (PMC3026821; doi:10.1371/journal.pone.0016442)
Supplement: Table S1 — IBD scoring. Values represent the mean ± SEM of IBD scores depicted in Figure 3a. (DOC) [file pone.0016442.s007.doc]

| **days** | **WT IBD Score** | **CCR9^-/-^ IBD Score** | **P value** |
| --- | --- | --- | --- |
| **d0** | 0.2500 ± 0.2500, N=4 | 0.1111 ± 0.1111, N=9 | 0.5614 |
| **d7** | 3.200 ± 0.8791, N=12 | 5.458 ± 0.8652, N=12 | 0.0807 |
| **d10** | 5.900 ± 1.260, N=10 | 6.950 ± 0.7550, N=10 | 0.4839 |
| **d13** | 2.400 ± 1.056, N=10 | 7.353 ± 0.9430, N=17 | ***0.0025*** |
| **d17** | 0.8000 ± 0.4701, N=15 | 6.333 ± 0.8980, N=15 | ***< 0.0001*** |

**Supplemental Table 1**

**IBD scoring.** Values represent the mean ± SEM of IBD scores depicted in Figure 3a.
