# Supplementary material for: CCL25/CCR9 Interactions Regulate Large Intestinal Inflammation in a Murine Model of Acute Colitis
Source: PLoS One. 2011 Jan 25;6(1):e16442. doi: 10.1371/journal.pone.0016442 (PMC3026821; doi:10.1371/journal.pone.0016442)
Supplement: Table S2 — Colon Length. Values represent the mean ± SEM of colon length depicted in Figure 3c. (DOC) [file pone.0016442.s008.doc]

| **days** | **WT Colon** | **CCR9^-/-^ Colon** | **P value** |
| --- | --- | --- | --- |
| **d0** | 8.831 ± 0.1591, N=13 | 8.555 ± 0.2906, N=11 | 0.5614 |
| **d7** | 6.669 ± 0.1482, N=13 | 6.160 ± 0.2891, N=10 | 0.0807 |
| **d10** | 6.679 ± 0.1948, N=14 | 6.490 ± 0.3716, N=10 | 0.4839 |
| **d13** | 7.023 ± 0.1613, N=26 | 6.959 ± 0.2156, N=17 | 0.0025 |
| **d17** | 7.635 ± 0.1017, N=31 | 6.809 ± 0.1364, N=23 | ***< 0.0001*** |

**Supplemental Table 2**

**Colon Length.** Values represent the mean ± SEM of colon length depicted in Figure 3c.
